# Analysis of age and gender associated *N*-glycoproteome in human whole saliva

**DOI:** 10.1186/1559-0275-11-25

**Published:** 2014-06-05

**Authors:** Shisheng Sun, Fei Zhao, Qinzhe Wang, Yaogang Zhong, Tanxi Cai, Peng Wu, Fuquan Yang, Zheng Li

**Affiliations:** 1 Laboratory of Functional Glycomics, College of Life Sciences, Northwest University, Xi’an 710069, P.R. China; 2 Laborotary of Proteomics, Institute of Biophysics, Chinese Academy of Sciences, Beijing 100101, P.R. China

**Keywords:** Saliva, Glycoproteome, Glycoproteins, Age, Gender, Hydrazide chemistry, Hydrophilic affinity, Mass spectrometry

## Abstract

**Background:**

Glycoproteins comprise a large portion of the salivary proteome and have great potential for biomarker discovery and disease diagnosis. However, the rate of production and the concentration of whole saliva change with age, gender and physiological states of the human body. Therefore, a thorough understanding of the salivary glycoproteome of healthy individuals of different ages and genders is a prerequisite for saliva to have clinical utility.

**Methods:**

Formerly *N*-linked glycopeptides were isolated from the pooled whole saliva of six age and gender groups by hydrazide chemistry and hydrophilic affinity methods followed by mass spectrometry identification. Selected physiochemical characteristics of salivary glycoproteins were analyzed, and the salivary glycoproteomes of different age and gender groups were compared based on their glycoprotein components and gene ontology.

**Results and discussion:**

Among 85 *N*-glycoproteins identified in healthy human saliva, the majority were acidic proteins with low molecular weight. The numbers of salivary *N*-glycoproteins increased with age. Fifteen salivary glycoproteins were identified as potential age- or gender-associated glycoproteins, and many of them have functions related to innate immunity against microorganisms and oral cavity protection. Moreover, many salivary glycoproteins have been previously reported as disease related glycoproteins. This study reveals the important role of salivary glycoproteins in the maintenance of oral health and homeostasis and the great potential of saliva for biomarker discovery and disease diagnosis.

## Background

Whole saliva is a slightly cloudy colorless liquid which is mainly comprised of the secretions of the parotid, submandibular, sublingual and minor salivary glands, and it exhibits multiple host defense functions in the maintenance of oral health [1]. Compared to other body fluids such as blood, cerebral spinal fluid (CSF) and urine, the collection of whole saliva is easy, noninvasive, safe, simple and cost-effective. Moreover, the significant overlap in protein content between saliva and plasma also suggests that saliva could be a potentially attractive fluid for disease biomarker discovery as well as a diagnostic alternative to blood tests [2,3]. However, a thorough understanding of whole saliva is a prerequisite for human saliva to have diagnostic utility.

For this reason, several salivary studies have focused on the proteomic composition of human whole saliva. In the published proteomic study of whole saliva, in 2004, Hu *et al.*[4] identified 64 non-redundant proteins using 2D-gel electrophoresis (2-DE) coupled with mass spectrometry (MS) analysis. In the following years, more advanced technologies for pre-separation and MS identification such as 2D-LC-MS/MS were used, and the numbers of identified salivary proteins increased to more than 1,500 [5-8].

The analysis of the salivary glycoproteome has also been conducted. Glycosylation is a common posttranslational modification which plays an important role in many cellular processes, e.g., protein conformation, folding, transport, targeting, and stability [9]. Many biological processes such as cell growth, differentiation, cell–cell communication, immune response, and microbial pathogenesis are also effected by glycosylation [10-13]. Moreover, glycosylation changes in glycoproteins have been identified in various diseases and can be used as biomarkers for disease diagnosis or prognosis [14,15]. Therefore, it is not surprising that there has been an increasing effort in applying glycoproteomic technologies to identify additional disease biomarkers from specific organs or in bodily fluids. These methods include lectin affinity, hydrazide chemistry, hydrophilic affinity, boronic acid affinity, size exclusion chromatography, and titanium dioxide-based enrichment [16-22]. Ramachandran, *et al.* was the first group to publish a study related to the salivary glycoproteome. They identified 84 formerly *N*-glycosylated peptides corresponding to 45 glycoproteins using the hydrazide capture technique coupled with mass spectrometry analysis [23]. Larsen *et al*. used the TiO_2_ enrichment strategy to isolate the whole salivary sialome, and 97 *N*-linked glycosylation sites were identified [20]. Ramachandran, *et al.* subsequently extended the salivary glycoprotein catalogue using the modified hydrazide capture method, and they identified a total of 156 formerly *N*-glycosylated peptides representing 77 unique *N*-glycoproteins in salivary fluid [24]. Most recently, using a novel hexapeptide library method, Bandhakavi, *et al*. significantly increased the number of salivary glycoproteins to 192 [25]. However, all these salivary *N*-glycoproteins were either from adult donors or the specific age and gender annotations were missing.

It is known that the composition of saliva changes with the physiological states of the human body [26]. The rate of production and the concentration of saliva differ before and after meals. Additionally, there are known differences in the production and concentration of saliva according to age and gender. It has been reported that the structure and function of salivary glands change with age and gender [27,28]. Animal research as well as human studies have revealed gender differences in the composition and production rate of saliva [29-31]. Moreover, women are more frequently affected by autoimmune diseases, such as Sjogrens syndrome and systemic lupus erythematosus, which affect salivary gland function [32]. Therefore, the first step toward the development of saliva diagnostic tests requires an understanding of the protein expression profiles of healthy individuals of different ages and genders.

In this study, two glycopeptide extraction methods, hydrazide chemistry and hydrophilic affinity, were employed coupled with mass spectrometry analysis to profile the whole salivary *N*-glycoproteomes of six different age and gender groups. The composition and biological functions of the whole salivary glycoproteomes among various age and gender groups were compared to determine differentiating trends. The results can facilitate an improved understanding of the significant functions of salivary glycoproteins in oral health and homeostasis as well as enhance the great potential of saliva for biomarker discovery and disease diagnosis.

## Results and discussion

### Protein concentrations and composition of human whole saliva

**Figure 1:**
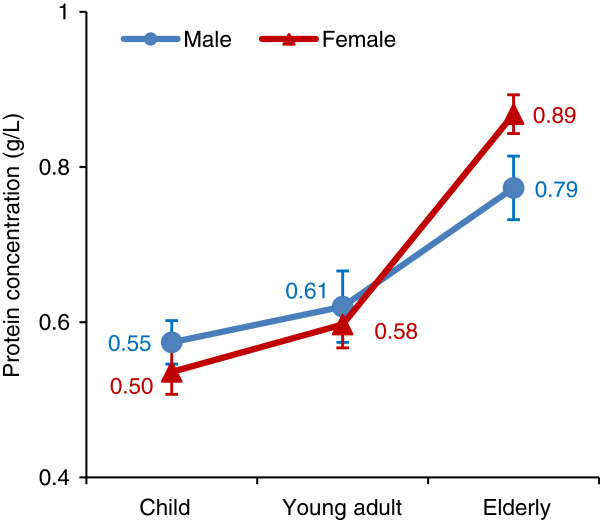
Comparison of protein concentrations of pooled whole saliva among different age and gender groups.

Six pooled saliva samples of different age and gender groups, including two gender groups of children (5–7 years old, mean age of 6.2 years, 28 males and 25 females), two gender groups of young adults (21–25 years old, mean age of 24.5 years, 13 males and 13 females) and two gender groups of the elderly (65–90 years of age, mean age of 71.7 years, 16 males and 14 females), were used for the study. After the collection and pooling of human whole saliva, the protein concentrations and composition of pooled saliva from the six age and gender groups were measured by BCA protein assay and SDS-PAGE, respectively. The overall trend was that the mean protein concentration of human whole saliva increased with age (Figure 1), in agreement with the results of a previous report [33]. The increasing rate was higher in females than in males. The increased age-related salivary protein concentration may be due to a combined effect of both the reduction of the production rate of whole saliva [34] and the increase of total protein content [35] as people age. The protein composition of saliva from different age and gender groups also showed a visible difference via gel electrophoresis (Additional file 1: Figure S1). The conspicuous differences of total protein content in human whole saliva among different ages and genders and the abundance of glycosylation on salivary proteins indicated the significance of the research regarding age- and gender-associated changes in the glycoproteome of human whole saliva.

### Identification of salivary *N*-glycoproteins

**Figure 2:**
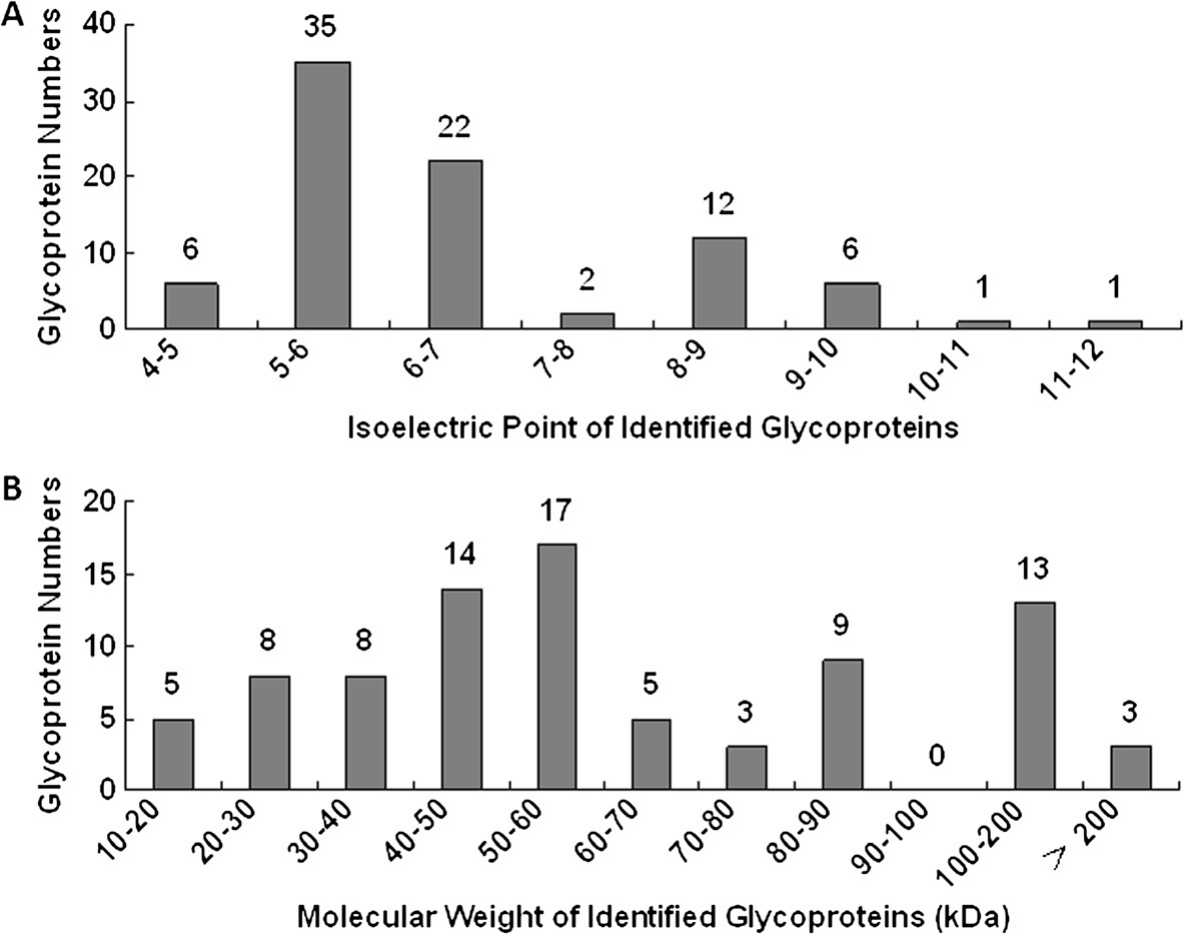
**Isoelectric point value (A) and molecular weight (B) distribution of *N-*** **glycoproteins and *N-*** **glycopeptides identified from human saliva.**

Two different methods, hydrazide chemistry and hydrophilic affinity, were employed for the isolation of formerly *N*-glycopeptide from six pooled saliva samples. To obtain a better understanding of the age-and gender-associated glycoprotein composition of whole saliva, an equivalent amount of salivary proteins (0.5 mg) from each group was used for *N*-linked glycopeptide isolation by each method. A total of 156 nonredundant formerly *N*-linked glycopeptides (*N*-deglycopeptides) representing 164 unique *N*-glycosylation sites and 85 *N*-linked glycoproteins were identified from human whole saliva by the two glycopeptide isolation methods (Additional file 2: Table S1). Of these, 119 glycopeptides (76%) from 70 glycoproteins (82%) were annotated as “known” *N*-glycopeptides in the UniProt database and 23 glycopeptides (15%) from 11 glycoproteins (13%) were annotated as “potential”. The other 14 *N*-glycopeptides (9%) were “novel” *N*-glycopeptides identified in this study (Additional file 2: Table S1, Additional file 3: Figure S2). After glycoprotein identification, some physiochemical characteristics of salivary glycoproteins, mainly their isoelectric point (pI) values and molecular weights (MWs), were statistically analyzed (Figure 2). The pI values of the identified salivary glycoproteins were between 4 and 12 (Figure 2A), with most clustering around 5–7, and 8–9. Approximately 3/4 (74.1%) of the identified salivary *N*-glycoproteins had pI values ≤7. The MW of most salivary *N*-glycoproteins (81.2%) ranged from 10-90 kDa and more than half (61.2%) ranged from 10-60 kDa (Figure 2B), which is similar to the MW distribution of the salivary proteome [3]. The results from the above analysis indicate that most salivary glycoproteins are acidic with low molecular weight.

### Comparison of salivary *N*-glycoproteomes among different age groups

To further understand the age-associated alterations of the human salivary *N*-glycoproteome, a comparison of human salivary *N*-glycoproteomes was conducted among different age groups after mass spectrometry identification. Note that for some glycoproteins, the differences among different age and gender groups detected in this study might just reflect the concentration changes of the glycoproteins in the whole saliva among age and gender groups.

**Figure 3:**
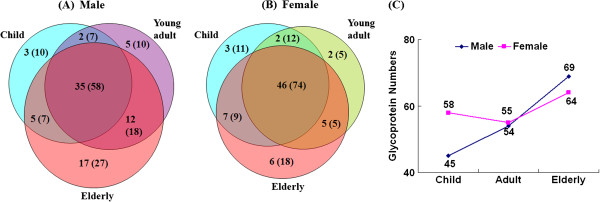
**Comparison of *N-*glycoproteins identified from whole saliva according to different age groups. (A)** Males; **(B)** Females; **(C)** Age-associated differences in the numbers of identified salivary glycoproteins.

Among the males, there were 45 (82), 54 (93), and 69 (110) *N*-glycoproteins (*N*-deglycopeptides) identified from the children, young adult, and elderly groups, respectively (Figure 3A). Thirty five *N*-glycoproteins were commonly identified in all three age groups; twelve, five and two glycoproteins were not identified in the children, young adult and elderly groups, respectively, while three, five, and seventeen *N*-glycoproteins were uniquely identified in the whole saliva of the children, young adult, and elderly groups, respectively.

In the female groups, the number of *N*-glycoproteins (*N*-deglycopeptides) identified from the children, young adult, and elderly groups was 58 (106), 55 (96), and 64 (106), respectively (Figure 3B). Of these, 46 *N*-glycoproteins were identified in all three age groups; five, seven and two glycoproteins were not identified in the children, young adult and elderly groups, respectively. Similarly, there were three, two, and six *N*-glycoproteins that were uniquely identified from the whole saliva of the children, young adult, and elderly groups, respectively. Based on these results, there was an increasing trend in the number of salivary *N*-glycoproteins that was associated with increasing age, and the increase in the number of *N*-glycoproteins was higher in the male groups than in the female groups, except for the female adult group which exhibited a small decrease compared to the female children group (Figure 3C).

The results also showed that eight salivary glycoproteins were likely to be associated with ageing (Table 1). For example, kallikrein-11 (KLK11) was only identified in the whole saliva of the male and female groups of children. Kallikrein-11 is a protein that belongs to a subgroup of serine proteases with diverse physiological functions [36]. Kallikreins are responsible for the coordination of various physiological functions including blood pressure, semen liquefaction and skin desquamation [37]. Golgi membrane protein 1 (GOLM1) was only identified in the whole saliva of the young adult groups. GOLM1 is a type II Golgi transmembrane protein. Its biological function is still unknown, but GOLM1 is hypothesized to be involved in the cellular response to viral infection [38]. Hypoxia up-regulated protein 1 (HYOU1), histidine-rich glycoprotein (HRG), and Olfactomedin-4 were only identified in the whole saliva of the elderly groups. HYOU1 belongs to the heat shock protein 70 family and it plays an important role in cytoprotective cellular mechanisms against hypoxia/ischemia-induced neuronal death [39,40]. HRG can bind several ligands such as heparin, heme, heparan sulfate, plasminogen, thrombospondin, and divalent metal ions. It can act as an adapter protein and it is involved in many biological processes such as immune complex formation and pathogen clearance, coagulation, angiogenesis, fibrinolysis, cell chemotaxis and cell adhesion. HRG mediates the clearance of necrotic cells by enhancing the phagocytosis of necrotic cells in a heparan sulfate-dependent pathway, and it is also involved in the regulation of tumor angiogenesis and tumor immune surveillance [41]. OLFM4 has been found to be up-regulated in gastrointestinal cancer [42] and many inflammatory diseases [43,44]. Biotinidase was not detected in the whole saliva of the groups of children, and two salivary *N*-glycoproteins, BPI fold-containing family B member 1 (BPIFB1) and UPF0762 protein C6orf58, were not detected in the elderly groups. BPIFB1 is a secreted protein which may play a role in innate immunity in the mouth, nose and lungs and it has a lipid binding function [45]. The biological function of UPF0762 protein C6orf58 remains unknown. In addition, biotinidase, which functions to catalyze biotin release from biocytin, was not detected in the groups of children. Based on this analysis, many salivary glycoproteins associated with ageing, particularly those that were uniquely identified or not identified in the elderly groups, are involved in the immune response and oral cavity protection. These results indicate the important role of salivary glycoproteins in the maintenance of oral health and homeostasis.

**Table 1:** Age- and gender-associated salivary glycoproteins

**Accession**	**Glycoprotein name**	**Age or gender group**
**Age-associated**		
IPI00002818	Isoform 1 of Kallikrein*-*11	Children
IPI00759659	Isoform 2 of Golgi membrane protein 1	Young adults
IPI00000877	Hypoxia up-regulated protein 1	Elderly
IPI00022371	Histidine-rich glycoprotein	Elderly
IPI00022255	Olfactomedin*-*4	Elderly
IPI00291410	Isoform 1 of Long palate, lung and nasal epithelium carcinoma-associated protein 1	Children, young adults
IPI00374315	UPF0762 protein C6orf58	Children, young adults
IPI00218413	Biotinidase	Young adults, elderly
**Gender-associated**		
IPI00006114	Pigment epithelium-derived factor	Male young adults, male elderly
IPI00006690	Eosinophil peroxidase	Male young adults, male elderly
IPI00305461	Inter-alpha (Globulin) inhibitor H2, isoform CRA_a	Female young adults, female elderly
IPI00029260	Monocyte differentiation antigen CD14	Female young adults, female elderly
IPI00291866	Plasma protease C1 inhibitor	All female groups, male elderly
IPI00478003	Alpha-2-macroglobulin	All female groups, male elderly
IPI00152154	Mucin*-*7	All female groups, male children

### Comparison of salivary *N*-glycoproteomes among different gender groups

**Figure 4:**
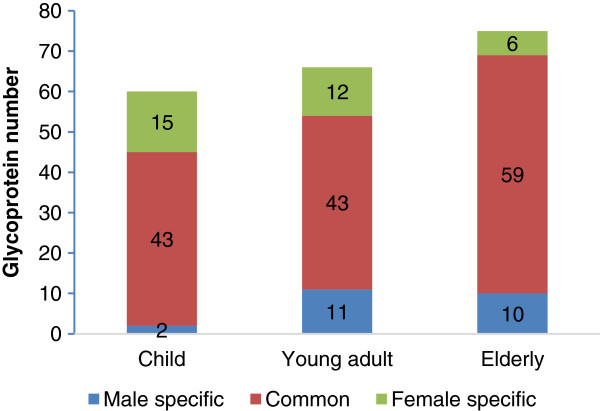
**Comparison of *N-*** **glycoproteins identified from whole saliva among different gender groups.**

A comparison of salivary *N*-glycoproteins (*N*-deglycopeptides) identified in different gender groups with the same age range is shown in Figure 4. Although children and young adult groups had same numbers (43) of common *N*-glycoproteins identified between males and females, the numbers of *N-*glycoproteins uniquely identified in the young adult group increased. The children group contained two unique *N*-glycoproteins that were specific to males and 15 that were specific to females, whereas the young adult group had 11 unique *N*-glycoproteins that were specific to males and 12 that were specific to females. Contrarily, compared to the young adult groups, the numbers of commonly identified *N*-glycoproteins between males and females increased in the whole saliva of the elderly groups, while the number of unique *N*-glycoproteins decreased slightly. There were 59 commonly identified *N*-glycoproteins between the male and female elderly groups, while 10 *N*-glycoproteins were uniquely identified in the male elderly group and six were uniquely identified in the female elderly group. These data indicated that the young adult groups had a slightly larger difference in the numbers of salivary *N*-glycoproteins between genders among the three age groups and the number of unique glycoproteins identified in the female groups decreased with age.

The results showed that some glycoproteins might also be associated with gender (Table 1). For example, Pigment epithelium-derived factor (PEDF) and eosinophil peroxidase (EPX) were only identified in the whole saliva of the male young adult and elderly groups. Inter-alpha (Globulin) inhibitor H2, isoform CRA_a and monocyte differentiation antigen CD14 (CD14) were identified in the female young adult and elderly groups. Plasma protease C1 inhibitor (SERPING1) was not identified in the male children and young adult groups and mucin 7 was not identified in the male young adult and elderly groups. Many of these glycoproteins, especially some female-specific glycoproteins, are also immune associated proteins. For example, CD14 mediates the innate immune response to bacterial lipopolysaccharide [46]; SERPING1 may potentially play a crucial role in regulating important physiological pathways including complement activation and blood coagulation [47]; A2M is involved in complement and coagulation cascade pathways [48]; and mucin-7 may have a protective capacity in promoting the clearance of bacteria in the oral cavity [49].With normal aging, the physiological states of the human body and microbial communities in the oral cavity may change significantly, which might cause changes in the salivary glycoproteome [23]. The majority of the proteins that were commonly identified in all six age and gender groups, including prolyl-rich protein, statherin, cysteine containing nitric acid protein, mucin, amylase, and salivary peroxidase are the basic protein components of saliva. Conversely, many proteins that were specifically expressed in different age and gender groups might function to regulate the physiological states of the human body and adapt to the specific microbial community in the oral cavity. Therefore, these age- and gender-associated glycoproteins might play a dominant role in the maintenance of oral health [1] and the immune response [23].

### Gene ontology (GO) analysis and disease association

**Figure 5:**
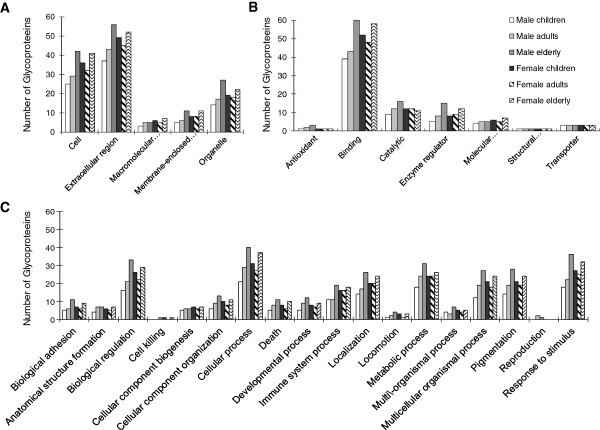
**Comparison of salivary *N*-glycoproteins among different age and gender groups based on Gene Ontology analysis of (A) cellular component (B) molecular function, and (C) biological process.**

To further study the biological function of the salivary glycoproteome in the maintenance of oral health, the salivary *N*-glycoproteins from different age and gender groups were further compared based on their GO annotation of cellular component, molecular function, and biological process terms (Figure 5). The majority of the salivary *N*-glycoproteins were located in the extracellular region (41%), cell (30%) and organelle (18%); thus, the number of glycoproteins from these cellular locations had the most markedly increasing trend corresponding with age, especially in males (Figure 5A). The GO distribution based on their biological functions showed that 62% of the salivary *N*-glycoproteins were involved in binding, 14% in catalytic activity and 13% in enzyme regulator activity. The numbers of salivary *N*-glycoproteins with these functions showed a noticeably increasing trend with age in the male groups. Conversely, the number of *N*-glycoproteins that function as structural molecules (IPI00855918) and transporters (IPI00022488, IPI00166729, IPI00299547) remained constant in all six different age and gender groups. The number of glycoproteins with molecular transducer activity also remained nearly constant among the different groups (Figure 5B).

With regard to biological processes (Figure 5C), the numbers of salivary *N*-glycoproteins belonging to most of the categories increased with age in the male groups, especially the *N*-glycoproteins that are involved in biological adhesion, biological regulation, cellular component organization, cellular process, death, development process, metabolic process, multicellular organismal process, pigmentation, and response to stimulus biological processes. Interestingly, one *N*-glycoprotein involved in cell killing (IPI00022395) and two proteins involved in reproduction (IPI00103633, IPI00006114) were also identified in the whole salivary proteome of adults and/or old males. Some exceptions to the age-related trend in the number of *N*-glycoproteins were determined. In particular, the numbers of salivary *N*-glycoproteins identified in the female young adult groups decreased slightly compared to the numbers identified in the female children, but the overall trend was also an increasing tendency with age. Of these, the number of immune-related glycoproteins increased with age in both male and female groups. However, in the younger age group, the number of immune, biological regulation, inhibition of enzyme activity and protein binding related salivary glycoproteins was significantly higher in females than in males. This difference was reduced with age and the level of these functional glycoproteins was largely similar between genders in the elderly group. These results may demonstrate the significant functions of salivary glycoproteins in oral health and homeostasis.

To assess the potential of salivary glycoproteins for disease biomarker discovery and diagnostic efforts, the association of salivary glycoproteins with human systematic diseases was also illustrated. Among the 85 proteins identified in the human whole saliva, 44 proteins (51.8%) were associated with human disease in a genetic association database and an OMIM disease database based on DAVID functional annotation (Additional file 4: Table S2). For example, nine glycoproteins including alpha-2-macroglobulin (IPI00478003) and myeloperoxidase (IPI00007244) are related to Alzheimer’s disease. Nineteen, 14, 17 and 15 salivary *N*-glycoproteins are associated with metabolic, neurological, immune, and cardiovascular diseases, respectively. These results indicate the great potential of saliva for biomarker discovery and disease diagnosis.

### Comparison of two glycopeptides isolation methods

A combination of hydrazide chemistry and hydrophilic affinity methods was used to increase the identification coverage of salivary glycoproteomes. Among 85 salivary N-glycoproteins (156 formerly N-glycopeptides) identified in the study, 43 N-glycoproteins (72 formerly N-glycopeptides) were identified by both methods, 39 N-glycoproteins (74 N-glycopeptides) were identified by hydrazide chemistry method uniquely, and 3 N-glycoproteins (10 N-glycopeptides) were identified uniquely by hydrophilic affinity method (Additional file 2: Table S1). Although some complementarity existed in two methods in the study, the hydrazide chemistry method showed higher specificity and identification rate of salivary N-glycoproteins than hydrophilic affinity method. This may be due to the inherent characteristic of these two methods: hydrazide resin captures glycoproteins/glycopeptides by covalent bonding, and thus non-specific adsorbed proteins can be thoroughly removed by intensely washing without any loss of glycopeptides. While Sepharose cell-4B captures glycoproteins/glycopeptides with hydrophilic interaction. The washing needs to be much mild, or many glycopeptides may be lost in the washing process. Besides, O-glycopeptides will also be released from Sepharose cell-4B in the elution process which may interfere with N-deglycopeptide identification by mass spectrometry. All the above reasons might result in the relatively low identification rate of the hydrophilic affinity method.

## Conclusion

In this study, the formerly *N*-linked glycopeptides of whole saliva were isolated, identified and compared among six different age and gender groups. The results showed that most salivary glycoproteins are acidic with low molecular weight. The number of salivary *N*-glycoproteins had an increasing age-associated trend, and the rate of increase was higher in the male groups than in the female groups. Fifteen salivary glycoproteins could be associated with gender or age. Based on their biological functions and gene ontology, many of the glycoproteins, especially those that were uniquely identified or not identified in the elderly groups, were involved in immune response and oral cavity protection. Moreover, more than half of the identified salivary glycoproteins were associated with human disease pathways. The data reveal the important role of salivary glycoproteins in the maintenance of oral health and homeostasis and the great potential of saliva for biomarker discovery and disease diagnosis.

## Materials and methods

### Human whole saliva collection

(1) Subject selection. Fifty-three healthy children (28 males and 25 females) between 5 and 7 years of age (mean age of 6.2 years), 26 young adults (13 males and 13 females) between 21 and 25 years of age (mean age of 24.5 years), and 30 elderly individuals (16 males and 14 females) between 65 and 90 years of age (mean age of 71.7 years) were selected from a primary school, our laboratory and a home for the aged. The subjects were randomized prior to their participation in the study. (2) Saliva collection. Whole, un-stimulated saliva was collected from subjects in the morning, 2 h after the last intake of food. The donors were asked to rinse their mouth with normal saline immediately before collection. The whole saliva was collected and placed on ice before being centrifuged at 12,000 rpm at 4°C for 60 min. The supernatant was collected and protease inhibitor (1 μL/ml whole saliva) was added to minimize protein degradation. Equal volumes of individual saliva samples were pooled to construct different age and gender pools that were as homogeneous as possible to reduce the individual variance, and the protein amount was measured using a BCA protein analysis kit (Pierce, Rockford, IL). The mixed saliva was stored at -80°C.

The collection of human whole saliva with informed consent was approved by the Human Ethics Committee of Northwest University and conducted in accordance with the ethical guidelines of the Declaration of Helsinki.

### SDS-PAGE

The mixed human whole saliva proteins were analyzed by SDS-PAGE. Ten μL of the samples were mixed with 2 × loading buffer, boiled for 5 min at 95°C, and applied onto a discontinuous 10% polyacrylamide gel. After SDS-PAGE, the bands on the gels were visualized via silver staining.

### Extraction and trypsin digestion of whole salivary proteins

Whole saliva containing 1 mg of proteins was concentrated by a 3kD Amicon Ultra centrifugal filter device (Millipore, Bradford, MA, USA) with denaturing buffer [50] (8 M urea in 0.1 M NH_4_HCO_3_ solution, pH 8.3) at 12,000 g for 20 min at 4°C (repeated three times) to exchange the solvent and denature the salivary proteins. The salivary proteins were then reduced by 5 mM DTT at 60°C for 60 min and alkylated by 20 mM iodoacetamide at room temperature in the dark for 30 min. The solution was diluted 5-fold with 0.1 M ammonia bicarbonate (pH 8.3) and the proteins were digested with 20 μg sequencing-grade modified trypsin (trypsin: protein, 1:50, w/w) overnight at 37°C. Digestion was terminated by acidifying the sample mixture with TFA to pH <3. The peptides were desalted by a Sep-Pak® Vac C18 cartridge (Waters, Milford, MA) and eluted in 0.4 mL of 80% ACN/0.1% TFA. The peptides were then divided into two equal aliquots. One aliquot was used for formerly *N*-glycopeptide isolation by the hydrazide chemistry method, while the other aliquot was used for formerly *N*-glycopeptide isolation by the hydrophilic affinity method.

### Formerly *N*-linked glycopeptide isolation using hydrazide chemistry

The *N*-glycopeptides of salivary proteins were enriched by the hydrazide method according to previously described protocols [51,52]. Briefly, half of the peptides (~0.5 mg) from each group were oxidized by 10 mM sodium periodate at room temperature for 1 h in the dark. The oxidized peptide samples were diluted 16-fold with 0.1% TFA and purified by C18 column. The peptides were eluted directly into hydrazide resin (Bio-Rad, Hercules, CA) and incubated overnight at room temperature with shaking.

The resin was washed three times each with 80% ACN, 1.5 M NaCl and D.I. water. The formerly *N*-linked glycopeptides were released from resin via 2 μL PNGase F (New England Biolabs, Beverly, MA) in 100 μl of 25 mM ammonium bicarbonate at 37°C overnight with shaking. The supernatant and wash solutions were combined and dried via SpeedVac.

### Glycopeptide enrichment by hydrophilic affinity method

The remaining half of the tryptic peptides (~0.5 mg) was mixed with agarose–hydrophilic resin (Sigma-Aldrich, St. Louis, MO) to bind the glycopeptides to the resin. After gently shaking for 45 min, the microcentrifuge tube was centrifuged (9,000 g, 5 min), and the resulting supernatant was removed. The resin was then washed three times with 80% ACN to remove non-glycosylated peptides. Finally, the glycopeptides bound to the resin were eluted twice with 200 μL H_2_O. The elution solutions were combined and were concentrated to ~50 μL by vacuum centrifugation. The *N*-glycans were removed from *N*-glycopeptides via 2 μl PNGase F overnight at 37°C with shaking in 25 μl of 100 mM ammonium bicarbonate solution. The solution was dried via SpeedVac.

### LC-MS/MS analysis

The lyophilized peptides were resuspended in 25 μL of 0.1% FA, and 8 μL was used for each LC-MS/MS run. LC-MS/MS analysis of peptides was conducted using an LC Packing nano-LC system (Agilent 1200 series) with a nanoelectrospray chip interface (Agilent) and a quadrupole TOF mass spectrometer (Agilent 6530 Accurate-Mass Q-TOF LC/MS, USA). The samples were first loaded onto an HPLC-Chip (G4240-62010, Zorbax 300SB C18 particles) for nano-LC separation at a flow rate of 300 nL/min. The eluents used for the LC were (A) 1% ACN/0.1% FA and (B) 90% ACN/0.1% FA. A gradient was utilized from 3% B to 10% B in 10 min, from 10% B to 45% B in 70 min, from 45% B to 95% B in 10 min, and held at 95% B for 15 min. Then the column was re-equilibrated for 15 min before the next run. Due to the statistical fluctuations of peptide precursor selection during the MS/MS acquisition, two LC-MS/MS assays were run for each sample to facilitate a proper proteome comparison.

### Data mining and analysis

Protein identification was accomplished utilizing the MASCOT database search engine (v.2.3.02, Matrix Science, London). The MS/MS spectra were used to search the IPI human 3.74 database in which trypsin and up to one miscleavage were specified. Carbamidomethylation (C) was set as a fixed modification, while oxidation (M) and deamination (N) were set as variable modifications. A peptide tolerance of 20 ppm and a tolerance of ± 0.7 Da for the fragment ions were used. The peptide identification was filtered by a Mascot Score above 30 with p < 0.05. *N*-Glycopeptide identification were filtered by a deamidated (N) site at N-X-S/T motif (X is any amino acid except Pro) to reduce potential false positive identification [53].

### Bioinformatic analysis of salivary glycoproteins

Gene Ontology (GO) analysis of the identified glycoproteins was conducted by Blast2GO [54], and comparative GO analysis between different age and gender groups was conducted by WEGO [55]. The whole analysis process was conducted according to standard operating procedures. The molecular weight and isoelectric point of the identified glycoproteins were obtained by MASCOT. Disease-associated salivary glycoproteins were identified by DAVID [56] functional enrichment analysis according to standard procedures.

## Competing interests

The authors declare that they have no competing interests.

## Authors’ contributions

SS, FZ, and ZL were involved in the study conception and design. FZ and YZ collected the saliva samples. FZ, QW, SS and YZ carried out the experiments. FY, TC, SS, and PW were involved in the mass spectrometry analysis. SS, FZ and ZL wrote the manuscript. SS, FZ and QW were involved in the data analysis and bioinformatic analysis. All authors read and approved the final manuscript.

## Supplementary Material

Additional file 1: Figure S1 SDS-PAGE analysis of human whole saliva from different age and gender groups. Line 1: Marker, Lines 2: pooled saliva of male children, Lines 3: pooled saliva of female children, Lines 4: pooled saliva of male young adults, Lines 5: pooled saliva of female young adults, Lines 6: pooled saliva of male elderly, Lines 7: pooled saliva of female elderly. Lines 2–7 were loaded with the same volume of pooled saliva. Click here for file

Additional file 2: Table S1 *N*-linked glycoproteins/glycopeptides and glycosylation sites identified from human whole saliva obtained from male and female patients of different ages. Click here for file

Additional file 3: Figure S2 UniProt-based annotation of *N*-glycopeptides and *N*-glycoproteins identified from human saliva. (A) *N*-glycopeptides. (B) *N*-glycoproteins. Click here for file

Additional file 4: Table S2 Disease associated glycoproteins identified in human whole saliva. Click here for file
