# Supplementary figures and images for: Analysis of age and gender associated N-glycoproteome in human whole saliva
Source: Clin Proteomics. 2014 Jun 5;11(1):25. doi: 10.1186/1559-0275-11-25 (PMC4070402; doi:10.1186/1559-0275-11-25)

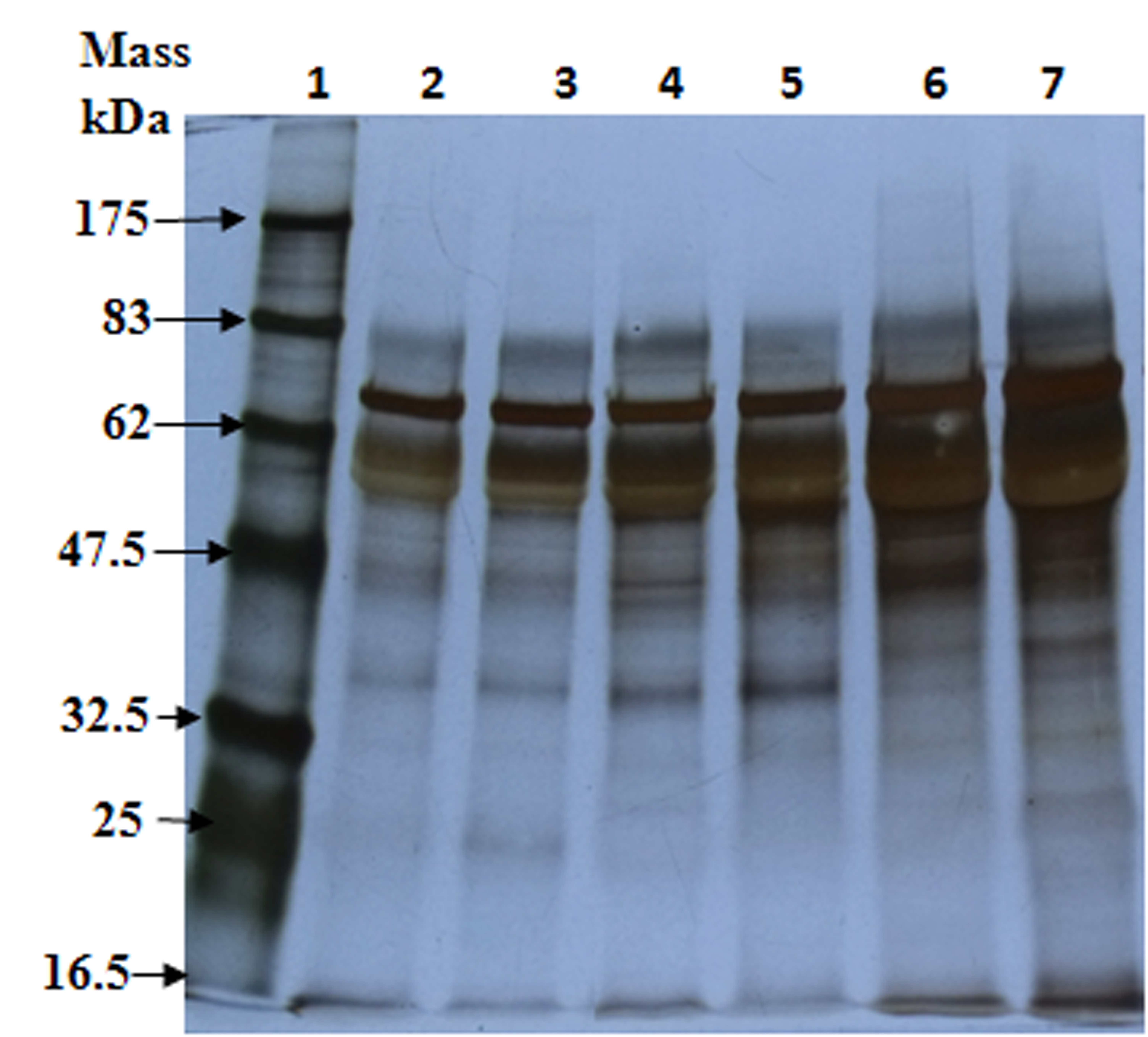

Supplement: Additional file 1: Figure S1 — SDS-PAGE analysis of human whole saliva from different age and gender groups. Line 1: Marker, Lines 2: pooled saliva of male children, Lines 3: pooled saliva of female children, Lines 4: pooled saliva of male young adults, Lines 5: pooled saliva of female young adults, Lines 6: pooled saliva of male elderly, Lines 7: pooled saliva of female elderly. Lines 2–7 were loaded with the same volume of pooled saliva. [file 1559-0275-11-25-S1.tiff]

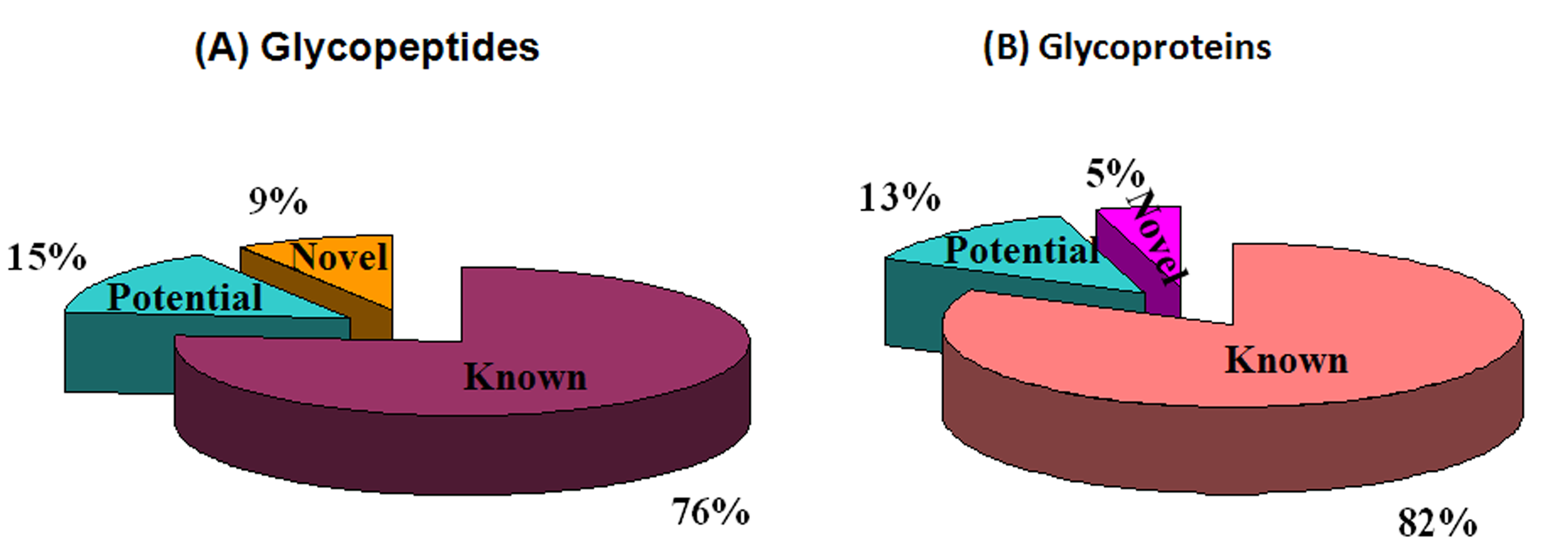

Supplement: Additional file 3: Figure S2 — UniProt-based annotation of N-glycopeptides and N-glycoproteins identified from human saliva. (A) N-glycopeptides. (B) N-glycoproteins. [file 1559-0275-11-25-S3.tiff]
